# A 10-Year Tertiary Care Center Experience With Adrenalectomies for Adrenal Tumors

**DOI:** 10.7759/cureus.21949

**Published:** 2022-02-06

**Authors:** Mohammad A Alghafees, Ziyad F Musalli, Khalaf Albaqami, Muhannad Q Alqirnas, Meshari A Alqahtani, Faisal Alrasheed, Ahmed Alasker

**Affiliations:** 1 College of Medicine, King Saud Bin Abdulaziz University for Health Sciences, Riyadh, SAU; 2 Urology, King Abdulaziz Medical City, Riyadh, SAU; 3 Medicine, King Abdullah International Medical Research Center, Riyadh, SAU

**Keywords:** saudi arabia, endocrine surgery, uro-oncology, adrenal tumours, adrenalectomies

## Abstract

Background

Due to limited data, our understanding of the trends and outcomes of adrenalectomy in the Saudi surgical practice is limited and insufficient. The aim of this study was to review the clinical data regarding the diagnosis and management of patients with adrenal masses and to assess the effect of surgeon specialty on the outcomes.

Materials and methods

The study included all adult patients who underwent an adrenalectomy for tumors from 2011 to 2021. The patient characteristics, tumor profile, and preoperative, perioperative, and post-operative variables were collected. Frequency and percentage were generated for the categorical variables, and mean and standard deviation were generated for the quantitative variables.

Results

A total of 55 patients were identified. Most of the patients had a well-defined (58.2%, n = 32), benign (85.5%, n = 47) mass located in the cortex (58.2% n = 32). The majority of the sample were asymptomatic (52.7%, n = 29), and the most frequent diagnosis was adrenal adenoma (47.27%, n = 26). The most frequent indication for surgery was tumor functionality (69.1%, n = 38). Surgeries were mostly laparoscopic (69.1% n = 38) and performed by a urologist (52.7%). The conversion to open surgery was 13%, the intraoperative complication rate was 9.1%, the post-operative complication rate was 7.3%, and the 30-day readmission rate was 3.6%. Intraoperative complications, post-operative complications, and conversion to open surgery were more frequent among general surgeons, while 30-day readmissions were more frequent among urologists. However, a statistically accurate association could not be found due to the small population size.

Conclusion

Open surgery was replaced by laparoscopic adrenalectomy as the surgery of choice for different adrenal pathologies. The findings reported in this study are substantiated by current literature, adding to our comprehension of adrenal tumor presentation. There are, however, some inconsistencies regarding the influence of gender on tumor development and the association between surgeon specialty and outcome in the literature that merit research. However, evidence regarding the contribution of comorbidities, such as hypertension, diabetes, and hypothyroidism, is lacking.

## Introduction

Adrenal tumors are the consequence of an abnormal proliferation of the adrenal gland cells. It can start in the cortex, the outer part of the adrenal gland, or the medulla, the inner part, and it can be benign or malignant. The majority of adrenal cortex tumors are benign and known as adenomas. Of the primary tumors of the adrenal gland, the most frequent primary adrenal tumor and the most frequent adrenal incidentaloma is adrenocortical adenoma (ACA) [1]. Adrenocortical carcinoma (ACC) is considered a rare endocrine cancer and has a five-year relative survival rate of 50% [2]. The annual incidence of ACC ranges from 0.5 to 2 per million persons globally. It is much less prevalent than cortical adenomas and pheochromocytoma, which affect the medulla. According to the American Cancer Society, adrenal adenomas are found in around one in every 10 people who had imaging, such as computed tomography (CT) or magnetic resonance imaging (MRI) of the adrenal gland. The adrenal gland is an endocrine organ that performs two tasks in the body. The cortex produces steroid hormones such as cortisol and mineralocorticoids (aldosterone, and the androgen dehydroepiandrosterone). The medulla produces catecholamines, including dopamine, epinephrine, and norepinephrine. Clinically, cortical tumors could be non-functioning or functioning tumors that produce aldosterone, cortisol, or sex hormones. Adrenal tumors are divided into two groups, according to the World Health Organization's (WHO) classification of endocrine tumors published in 2017. The first category includes tumors of the adrenal cortex, and, the second, tumors of the adrenal medulla and extra-adrenal paraganglia [3]. For the types of tumors, primary ACA, adrenal myelolipoma (mesenchymal and stromal tumors), and adrenal adenomatoid tumors are benign tumors of the adrenal cortex. Most primary ACAs are non-functioning adenomas, and ACC is a primary cancerous tumor of the adrenal gland. Approximately half of the ACCs are non-functioning. Cortisol-producing ACC is considered the most frequent. It has a bimodal age distribution with a female predominance [4]. Hematological tumors, which include lymphomas and plasmacytomas, are another type of primary tumor. The majority of the secondary tumors are metastatic lesions caused by direct infiltration or, more typically, hematogenous dissemination, usually in advanced cancer patients. The most frequent primary tumor of the adrenal medulla is pheochromocytoma [5]. Most pheochromocytomas have an equal sex distribution and occur in the fifth decade [4]. Approximately 800 cases are diagnosed annually in the United States [2], of which 30% are hereditary. It is considered the most prevalent hereditary tumor in humans [5]. Globally, a retrospective study conducted in China with 1,287 patients diagnosed with adrenal tumors reported that the median age of diagnosis was 62 years, with a female majority. Only 1% were younger than 18 years at diagnosis. A malignant adrenal tumor was diagnosed in 8.7% of patients, four (0.3%) had ACC, and 108 (8.4%) had other malignancies. Benign adrenal tumors were diagnosed in 1,175 (90%) patients, and 1,076 (83.6%) patients were diagnosed with ACA. Of the 1,076 adrenal adenomas, 53 (4.9%) had overt excess hormones, 140 (13%) were non-functioning adrenal adenomas, and 88 (8.2%) were mild autonomous cortisol secretion. However, the hormonal workup was incomplete in 795 (73.9%) [6]. An Italian study assessed adrenal tumors from a surgical perspective. The study included 68 patients who underwent bilateral or unilateral laparoscopic adrenalectomy. Half (n = 34) of the patients had adrenal masses, which was discovered incidentally, and 52% of these adrenal masses were benign adrenal adenomas, and only one (0.2%) was ACC. Just less than half (n = 30) were diagnosed with functioning tumors, 15 (50%) of which were pheochromocytomas, five (16%) were ACAs, and two (0.6%) were ACCs. Removal was complete in five of 77 lesions and partial (sparing adrenalectomy) in one patient with bilateral pheochromocytoma [7]. In a similar study in India including 17 patients who underwent laparoscopic adrenalectomy, 10 (58.82%) patients were diagnosed with functioning adrenal masses and only seven (41%) had non-functioning tumors. In the pathological examination, ACA was found in nine (52.94%) patients, bilateral pheochromocytoma in two (11.76%) patients, myelolipoma in two patients, of which one was converted to open surgery (11.76%), and adrenocortical hyperplasia in two (11.76%) patients. According to the study, laparoscopic adrenalectomy is a safe and effective minimally invasive treatment option for both functioning and non-functioning adrenal masses [8]. Locally, to our knowledge, only one study was conducted on the same topic, which investigated the indications of adrenalectomy and compared open and laparoscopic adrenalectomy. The study was conducted from 1999 to 2002 in Riyadh, Saudi Arabia. In total, 21 adrenalectomies were performed in 19 patients: nine by open procedure (seven patients) and 12 laparoscopic (12 patients). The indications for adrenalectomy were pheochromocytomas in five (23%) patients, Conn’s adenoma in 6 (28%) patients, Cushing’s disease in three (14%) patients, and ACC in only one (0.4%) patient. The study concluded that laparoscopic adrenalectomy is a safe procedure for most adrenal pathology [9]. Our understanding of the prevalence of adrenal tumors, incidence rate, types, treatment methods, and age group in Saudi Arabia is limited and insufficient. The aim of this study was to provide a 10-year experience with adrenalectomies of adrenal tumors in King Abdulaziz Medical City (KAMC), Riyadh, Saudi Arabia.

## Materials and methods

This retrospective chart review study was conducted at KAMC. All the patients in KAMC who underwent an adrenalectomy for an adrenal tumor from August 2011 to August 2021 were included. Patients who underwent the procedure elsewhere and only followed up at KAMC were excluded. The electronic records in the BESTCare system (ezCareTech, Seoul, South Korea) and paper records were reviewed. The following variables were extracted: age, gender, comorbidities, body mass index (BMI), presenting signs and symptoms, diagnosis, indication for adrenalectomies, diagnosis to intervention interval, basis of diagnosis, surgery type, surgeon’s specialty, tumor laterality, American Society of Anesthesiologists (ASA) score, preoperative blood pressure, number of hypertension medications, types of hormones secreted, operative time, estimated intraoperative blood loss, intraoperative blood transfusion, conversion to open surgery, post-operative complications, hospital length of stay, 30-day readmission, morphology, behavior, origin site, and size.

The data were entered in Microsoft Excel 2019 (Microsoft Corporation, WA, USA) and analyzed using the Statistical Package for the Social Sciences (SPSS) version 23.0 (IBM Corp., Armonk, NY, USA). Frequency and percentage were produced for the categorical variables, and mean and standard deviation (SD) for the quantitative variables.

The study was approved by the Institutional Review Board of King Abdullah International Medical Research Center, Ministry of National Guard - Health Affairs, Riyadh, Saudi Arabia (approval number NRC21R/351/07). Serial numbers were used instead of the medical record number to ensure confidentiality. Due to the retrospective nature and the use of anonymized patient data, the requirement for consent was waived.

## Results

In total, 55 patients were included. Table 1 shows the demographic profile. Less than half (43.6%, n = 24) were males, with 31 (56.4%) females. In terms of the BMI, 11 (20%) of the patients had a normal weight, 19 (34.5%) were overweight, and 25 (45.5%) were obese. The mean age was 49.91 years (SD: 15.10). The mean systolic blood pressure was 143 mmHg (SD: 21), and the mean diastolic blood pressure was 82.5 mmHg (SD: 16.3).

**Table 1:** Patients’ Profile (n = 55)

Demographical Characteristics	
Gender	
Male, n (%)	24 (43.60)
Female, n (%)	31 (56.40)
Body mass index (BMI)	
Normal weight, n (%)	11 (20.00)
Overweight, n (%)	19 (34.50)
Obesity, n (%)	25 (45.50)
Age	
Mean	49.91
Standard deviation	15.10
Blood pressure	
Systolic: mean (standard deviation)	143 (21)
Diastolic: mean (standard deviation)	82 (16)

**Figure 1:**
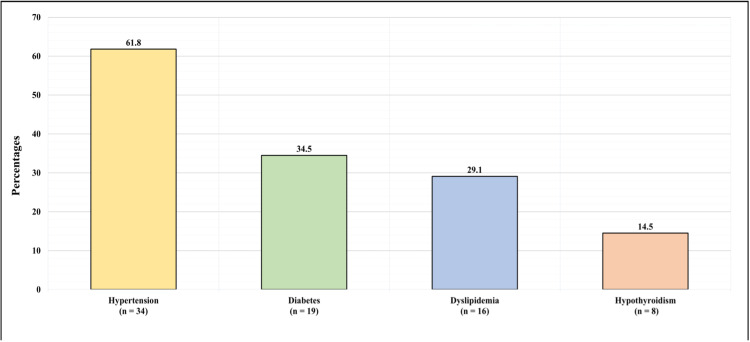
Patients' Medical History

Figure 1 shows the medical history of the patients. The majority (61.8%, n = 34) of the patients had hypertension, 19 (34.5%) had diabetes, 16 (29.1%) had dyslipidemia, and 8 (14.5%) had hypothyroidism. Regarding hypertension medication, 16 (29.1%) of the patients were not taking any hypertensive medication, eight (14.5%) were taking one antihypertensive medication, 17 (30.9%) were taking two antihypertensive medications, 11 (20%) were taking three antihypertensive medications, one (1.82%) was taking four antihypertensive medications, one (1.82%) was taking five antihypertensive medications, and one (1.82%) was taking six antihypertensive medications.

Table 2 presents the adrenal tumor profile. In terms of the diagnosis, 26 (47.27%) of the patients had adrenal adenoma, 17 (30.91%) had pheochromocytomas, three (5.45%) had myelolipoma, two (3.64%) had adrenal cystic tumor, one (1.82%) had adrenal cortical carcinoma, one (1.82%) had ganglioneuroma, one (1.82%) had paraganglioma, and four (7.27%) had multiple adrenal tumors. Of the group with multiple tumors, all four had pheochromocytoma, two (3.64%) had paraganglioma, one (1.82%) had ganglioneuroma, and one (1.82%) had a para-aortic ganglioma. Regarding the tumor morphology, 23 (41.8%) had an ill-defined morphology, and 32 (58.2%) had a well-defined morphology. In the majority (85.5%, n = 47) of the patients, the tumor was benign, and it was malignant in eight (14.5%). The origin of the tumor was in the medulla of 23 (41.8%) of the patients and in the cortex of 32 (58.2%) of the patients. The majority of the patients were asymptomatic (52.7%, n = 29). However, the most frequently observed signs and symptoms were hypertension (16.4%, n = 9), headache (10.6%, n = 6), and abdominal pain (7.3%, n = 4). In terms of the tumor dimensions, the mean height was 5.27 cm (SD: 3.64), the mean depth of tumor was 3.72 cm (SD: 2.54), and the mean width was 2.6 cm (SD: 2).

**Table 2:** Adrenal Tumor Profile (n = 55)

Adrenal Tumor Profile (n = 55)		
Question	n	%
Diagnosis		
Adrenal adenoma	26	47.27
Pheochromocytoma	17	30.91
Myelolipoma	3	5.45
Adrenal cystic tumor	2	3.64
Adrenal cortical carcinoma	1	1.82
Ganglioneuroma	1	1.82
Paraganglioma	1	1.82
Patients with multiple tumors, n = 4 (7.27%)		
Pheochromocytoma and paraganglioma	2	3.64
Pheochromocytoma and ganglioneuroma	1	1.82
Pheochromocytoma and para-aortic ganglioma	1	1.82
Morphology		
Ill defined	23	41.8
Well defined	32	58.2
Behavior		
Benign	47	85.5
Malignant	8	14.5
Origin		
Medulla	23	41.8
Cortex	32	58.2
Signs and symptoms		
Asymptomatic	29	52.7
High blood pressure	9	16.4
Headache	6	10.9
Abdominal pain	4	7.3
Flank pain	3	5.5
Hematuria	2	3.6
Hypokalemia	2	3.6
Multiple fractures	1	1.8
Fatigue	1	1.8
Weakness	1	1.8
Nausea	1	1.8
Vomiting	1	1.8
Diarrhea	1	1.8
Ecchymosis	1	1.8
Back pain	1	1.8
Weight loss	1	1.8
Sweating	1	1.8
Palpitation	1	1.8
Numbness	1	1.8
Tumor dimensions		
Height, mean (SD)	5.27 (3.64) centimeters	
Depth, mean (SD)	3.72 (2.54) centimeters	
Width, mean (SD)	2.60 (2.00) centimeters	

**Figure 2:**
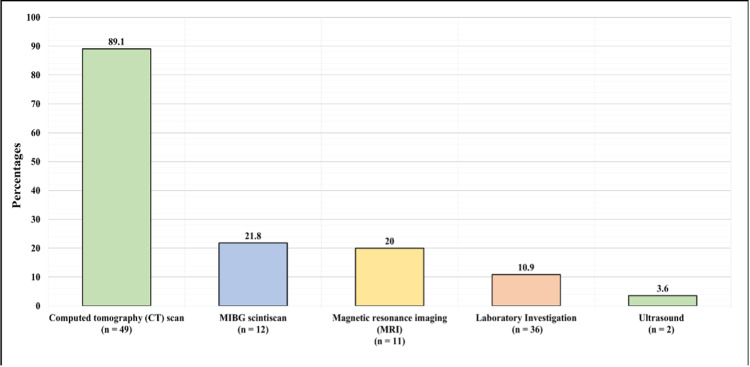
Basis of Diagnosis

Figure 2 demonstrates the basis of the diagnosis. The basis of the diagnosis was CT scan in 49 (89.1%) of the patients, MIBG (metaiodobenzylguanidine) scintiscan in 12 (21.8%), MRI in 11 (20%), laboratory investigations in 36 (10.9%), and ultrasound in two (3.6%).

**Figure 3:**
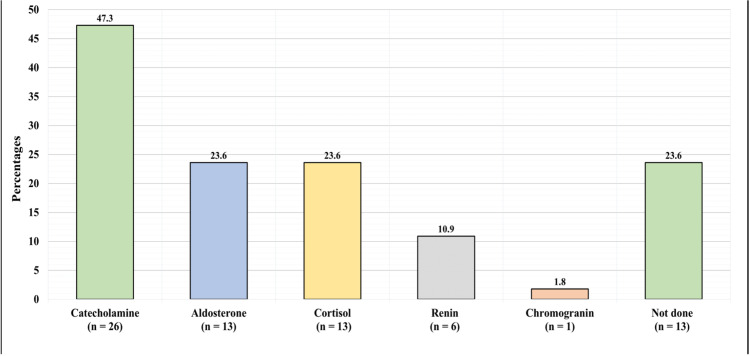
Preoperative Hormonal Panel

Figure 3 shows the preoperative hormonal panel. The preoperative hormonal panel was not performed for 13 (23.6%) of the patients. However, it revealed a high level of catecholamine in 26 (47.3%) of the patients, high aldosterone in 13 (23.6%), high cortisol in 13 (23.6%), high renin in six (10.9%), and high chromogranin in one (1.82%).

Table 3 shows the adrenalectomy profile. The indication of adrenalectomy was large in size in 17 (30.9%) of the patients and functional in 38 (69.1%) of the patients. As for the lateralization, the tumor was in the left side in 26 (47.3%) patients, right side in 28 (50.9%), and bilateral in one (1.8%). Transabdominal open surgery was performed in nine (16.4%) of the patients, transabdominal robotic in eight (14.5%), and lateral transabdominal laparoscopic in 38 (69.1%). The operating surgeon specialty was general surgery in 26 (47.3%) of the patients and urology for in (52.7%). Six (13%) of the patients who initially had a non-open approach had a conversion to open surgery. Two (4.3%) of the conversions were made by the urologists and four (8.7%) by the general surgeons. Intraoperative complications occurred in five (9.1%) of the patients, including air embolism in one (1.82%) and hemorrhage in four (7.27%). Of the group with a hemorrhage, one (1.82%) hemorrhaged from the renal hilum, which required a left nephrectomy, one (1.82%) with excessive bleeding required a blood transfusion, and two (3.63%) minimal bleeding that did not require blood transfusion. A small proportion (7.3%, n = 4) of the patients had a post-operative complication (hypertension). Only two (3.6%) patients required readmission within 30 days after the operation. The mean diagnosis to intervention interval was 330.51 days (SD: 527.81), and the overall mean operative time for all approaches was 3.79 hours (SD: 1.15). The mean estimated blood loss was 386.75 mL (SD: 350.76). The mean hospital length of stay was 8.67 days (SD: 5.54).

**Table 3:** Adrenalectomy Profile (n = 55) ASA, American Society of Anesthesiologists

Adrenalectomy Profile (n = 55)		
Question	n	%
Indication for adrenalectomy		
Large size	17	30.90
Functional	38	69.10
Lateralization		
Left	26	47.3
Right	28	50.9
Bilateral	1	1.8
Type of surgery		
Transabdominal open	9	16.4
Transabdominal robotic	8	14.5
Transabdominal laparoscopic	38	69.1
Surgeon specialty		
General surgery	26	47.3
Urology	29	52.7
Conversion to open surgery in patients with initially non-open approach (n = 46)		
Conversion to open surgery	6	13
Intraoperative complications		
No	50	90.9
Yes	5	9.1
Post-operative complications		
No	51	92.7
Yes	4	7.3
Readmission within 30 days post-op		
No	53	96.4
Yes	2	3.6
Diagnosis to intervention interval, mean (SD)	330.51 (527.81) days	
ASA score, mean (SD)	2.61 (0.63)	
Operative time, mean (SD)	3.79 (1.15) hours	
Estimated blood loss, mean (SD)	368.75 (350.76) milliliters	
Hospital length of stay, mean (SD)	8.67 (5.54) days	

Table 4 shows the frequency of different outcomes across surgeon specialty. Intraoperative complications, post-operative complications, and conversion to open surgery were more frequent among general surgeons, while 30-day readmissions were more frequent among urologists. However, a statistically accurate association could not be found due to the small population size.

**Table 4:** Frequency of Different Outcomes Across Surgeon Specialty

	Surgeon Specialty	
	Urology	General Surgery
Intraoperative complication, n (%)	2 (6.9%)	3 (11.5%)
Post-operative complication, n (%)	1 (3.4%)	3 (11.5%)
Conservation to open surgery, n (%)	2 (6.9%)	4 (15.4%)
30-day readmission, n (%)	2 (6.9%)	0

## Discussion

Patient demographics

The current findings suggest that adrenal tumors may be more prevalent in females than males (a female:male ratio of 1:1.29). The mean age of the participants was 49.91 years. The male-to-female ratio is not consistent with the literature. Sisman et al. conducted a single-center study to evaluate the contribution of the patient and treatment-related factors to the prognosis in ACC in 15 patients. The male-to-female ratio was 1:3, with the females being the primary source of diagnosis [10]. Parianos et al. reported a 20-year experience of adrenocortical cancer at a tertiary hospital, reporting a male-to-female ratio of 1:1.37, more consistent with our finding [11]. The median age, however, was consistent with our findings at 52.45 years, as reported by Sisman et al., and 55.5 years, as reported by Parianos et al [10,11]

The majority of the patients were overweight (34.5%) or obese (45.5%), suggesting a relationship between BMI and the development of adrenal tumors. This corresponds with the patients’ medical history, with the most prevalent comorbidities being hypertension, diabetes, dyslipidemia, and hypothyroidism. Although few studies reported the prevalence of these conditions in adrenal cancer patients, Kolańska et al. found a strong association between obesity and non-functioning adrenal tumors in 143 patients, with a 40% prevalence of obesity in the study group [12]. The literature also estimates that 60% of adrenal cancer patients present with symptoms of Cushing’s syndrome, characterized by weight gain, hypertension, and hyperglycemia [13].

High catecholamine, aldosterone, cortisol, and chromogranin levels were observed in the group with a preoperative hormone panel. Similar findings are reported frequently in the literature, with primary aldosteronism, hypercortisolism, and androgen excess being regularly reported [14,15] However, it is suggested that tumor morphology is a better predictor of metastatic risk than hormonal panels [16].

Adrenal tumor profile

The most prevalent tumor profiles in our cohort were adrenal adenoma, pheochromocytoma, and myelolipoma. More than half of the sample (58.2%) had a well-defined tumor morphology. These findings were loosely replicated in a previous study, with adenoma being the most prevalent histopathologic type (43.14%), followed by metastatic adrenal gland tumor (15.68%) and pheochromocytoma (12.75%) [10]. Tumor behavior was benign in the majority of the group presenting with adrenal masses (85.5%), also supported in the literature. A population-based study conducted by Ebbehoj et al. reported that in a cohort of 1,287 patients, 90.3% of adrenal tumors were benign [14]. The tumor origin was the cortex or the medulla (58.2% and 41.8%, respectively). Sisman et al., however, reported the origin of the patient tumors as a primary or a metastatic tumor (80.39% and 15.69%, respectively) [10].

Adrenalectomy profile

The lateralization of the tumor was equal, with the right side slightly higher (50.9% vs 47.3%), and bilateral in one patient, consistent with the literature. Sisman et al. reported 46.7% of the tumors on the right side and 53.3% on the left side [10]. Laparoscopic surgery was the most frequent intervention performed in the current study, consistent with the recommendations in the literature. Minimally invasive laparoscopic adrenalectomy remains the gold standard for benign adrenal diseases [16]. Despite this, complications, including an air embolism, bleeding, and post-operative hypertension, were reported in our cohort. However, the incidence was significantly lower than that reported in other studies. Suzuki et al. noted that 32% of their 21-patient cohort experienced complications, including vascular injury, organ injury, and massive bleeding [17].

Association between surgeon specialty and outcomes of adrenalectomy

Intraoperative complications, post-operative complications, and conversion to open surgery were more frequent among general surgeons, while 30-day readmissions were more frequent among urologists. However, a statistically accurate association could not be found due to the small population size.

Fuletra et al. exclusively investigated the association between 3,358 patient outcomes following adrenalectomy and the surgical speciality performing the procedure. General surgeons performed 90% of the procedures (n = 3,012) and urologists performed 10% (n = 334). The outcomes measured included the number of post-surgical complications, length of stay, rate of reoperation, 30-day readmission, and mortality. No statistically significant differences were observed between the general surgeons and the urologists [18]. The literature also corroborates that adrenalectomy is more frequently performed by general surgeons compared to urologists. In a population-based retrospective analysis conducted by Park et al., a significantly higher proportion of adrenalectomies were conducted by general surgeons as opposed to urologists [19]. Moreover, some reports state that the outcome is not only dependent on the surgeon but on the whole team involved in perioperative care where centers with a higher volume proved to have better outcomes [20]. Also, gaining the experience with laparoscopic adrenalectomy was proven to reduce complication rate, shorten length of hospital stay, and be more cost-effective, implying that surgeon experience is more important than the specialty [20,21]

Limitations

There were some limitations to the current study. First, due to its retrospective nature, the research team had to rely on others for accurate and safe recordkeeping. Some data may be missing from the BESTCare system or the paper records. Secondly, the population size is relatively small compared to larger multicenter studies in the literature.

## Conclusions

The findings reported in this study are generally substantiated by the literature, adding to our comprehension of adrenal tumor presentation. Adrenal masses are uncommon in younger patients and primarily affect males and females equally. There are, however, some inconsistencies regarding the influence of gender on tumor development in the literature that merit additional research. The role of hormonal changes is irrefutable, with a high level of catecholamine, aldosterone, cortisol, and chromogranin levels noted in the sample and similar cohorts in the literature.

Regarding the adrenal tumor profile, the most prevalent were adrenal adenoma, pheochromocytoma, and myelolipoma, with the tumor behavior in the vast majority of patients being benign. Lateralization was equal in terms of right and left, with only one patient presenting with a bilateral mass. Intraoperative complications, post-operative complications, and conversion to open surgery were more frequent among general surgeons, while 30-day readmissions were more frequent among urologists. However, a statistically accurate association could not be found due to the small population size. Despite the link between BMI and the development of adrenal tumors widely accepted in the healthcare industry, there is limited information available regarding the pathophysiology. In addition, evidence regarding the contribution of comorbidities, such as hypertension, diabetes, and hypothyroidism, is lacking.
